# Antinociceptive effect of intermittent fasting via the orexin pathway on formalin-induced acute pain in mice

**DOI:** 10.1038/s41598-023-47278-3

**Published:** 2023-11-20

**Authors:** Hyunjin Shin, Jaehyuk Kim, Sheu-Ran Choi, Dong-Wook Kang, Ji-Young Moon, Dae-Hyun Roh, Miok Bae, Jungmo Hwang, Hyun-Woo Kim

**Affiliations:** 1 Department of Physiology and Medical Science, College of Medicine and Brain Research Institute, Chungnam National University, 266 Munhwa-ro, Jung-gu, Daejeon, 35015 Korea; 2 CNS Team, N-DIC, Hwaseong, 18469 Korea; 3 Department of Pharmacology, Catholic Kwandong University College of Medicine, Gangneung, 25601 Korea; 4 Animal Protection and Welfare Division, Animal and Plant Quarantine Agency, Gimcheon, 39660 Korea; 5 Department of Oral Physiology, School of Dentistry, Kyung Hee University, Seoul, 02447 Korea; 6 Preclinical Research Center, Chungnam National University Hospital, Daejeon, 35015 Korea; 7 Department of Orthopaedic Surgery, College of Medicine, Chungnam National University, 266 Munhwa-ro, Jung-gu, Daejeon, 35015 Korea

**Keywords:** Somatosensory system, Preclinical research

## Abstract

It has been suggested that stress responses induced by fasting have analgesic effects on nociception by elevating the levels of stress-related hormones, while there is limited understanding of pain control mechanisms. Here, we investigated whether acute or intermittent fasting alleviates formalin-induced pain in mice and whether spinal orexin A (OXA) plays a role in this process. 6, 12, or 24 h acute fasting (AF) and 12 or 24 h intermittent fasting (IF) decreased the second phase of pain after intraplantar formalin administration. There was no difference in walking time in the rota-rod test and distance traveld in the open field test in all groups. Plasma corticosterone level and immobility time in the forced swim test were increased after 12 h AF, but not after 12 h IF. 12 h AF and IF increased not only the activation of OXA neurons in the lateral hypothalamus but also the expression of OXA in the lateral hypothalamus and spinal cord. Blockade of spinal orexin 1 receptor with SB334867 restored formalin-induced pain and spinal c-Fos immunoreactivity that were decreased after 12 h IF. These results suggest that 12 h IF produces antinociceptive effects on formalin-induced pain not by corticosterone elevation but by OXA-mediated pathway.

Any kind of change that puts physical, mental, or psychological pressure on a person is considered to be stressful^1^. Our body's reaction to anything that demands focus or action is stress, which is classified into three types: acute stress, episodic stress, and chronic stress. Fear, concern, difficulty relaxing, increased heart rate, breathing problems, disruption of sleep, change in eating habits, difficulty focusing, worsening of pre-existing health disorders (physical and mental), and increasing use of alcohol, cigarettes, and other substances are all signs of stress^2^. Stress has impacts on the brain as well as body^3^. It is well established that stress increases the activity of hypothalamus–pituitary–adrenal (HPA) axis resulting in the excessive secretion of corticosterone and noradrenalin from adrenal cortex^4^. Thus, corticosterone and noradrenalin levels are used as biomarkers for stress^5^.

Corticosterone is a stress hormone produced in the adrenal glands that has been shown to have analgesic effects through its interaction with the nervous system^6^. Corticosterone can activate the analgesic pathway in the brain by binding to glucocorticoid receptors, which are present in various regions of the central nervous system including the hypothalamus, hippocampus, and amygdala^7^. This activation leads to the release of endogenous opioids, such as enkephalins and beta-endorphins, in the brain^8^. Endogenous opioids are natural pain-relieving chemicals that bind to opioid receptors in the brain and spinal cord, and their release can lead to a reduction in pain perception^9^. Corticosterone-induced activation of the analgesic pathway can therefore result in pain relief.

Orexin, also known as hypocretin, is a neuropeptide synthesized and released from hypothalamic neurons^10^. It plays an important role in regulating various physiological processes including wakefulness, appetite and energy homeostasis^11–14^. Recent studies have shown that orexin A (OXA) binds to orexin receptors in various areas of the brain, including the hypothalamus, amygdala, and periaqueductal gray (PAG) area^15^. Activation of orexin receptors in the PAG region leads to the release of endogenous opioids such as beta-endorphin in the brain. Overall, OXA-induced analgesia in the mouse brain involves the activation of opioid pathway and the modulation of other neurotransmitter systems, leading to a reduction in pain perception^16^.

The analgesic effects of corticosterone and fasting are believed to involve complex interactions between multiple physiological pathways, including those related to pain processing, metabolism, and stress response^17^. In the present study, we trained mice to become accustomed to fasting-induced stress and investigated whether this programmed fasting alleviates formalin-induced inflammatory pain and whether OXA contributes to the modulation of pain in this process.

## Results

### Acute or intermittent fasting reduces phase 2 nociceptive behaviors in the formalin test

**Figure 1:**
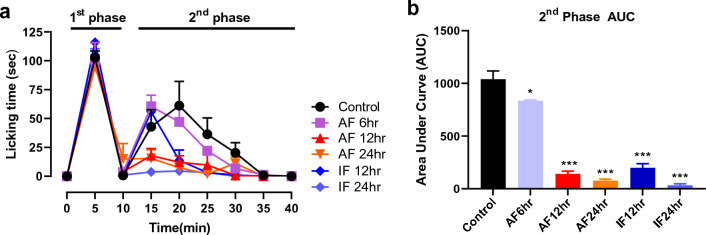
Effects of acute and intermittent fasting on formalin-induced pain behaviors. (**a**) Formalin-induced pain behaviors were measured for 40 min at 5-min intervals after 6 h acute fasting (AF 6 h), 12 h acute fasting (AF 12 h), 24 h acute fasting (AF 24 h), 12 h intermittent fasting (IF 12 h), and 24 h intermittent fasting (IF 24 h). Licking behaviors were classified as 1st phase (0–10 min) and 2nd phase (10-40 min). (**b**) Area under curve (AUC) in the 2nd phase of pain was analyzed in control and fasting groups. Data were expressed as the mean ± SEM. *p < 0.05 and ***p < 0.0001 vs. Control. n = 7 mice/group.

To investigate the effect of fasting on inflammatory pain behaviors, we administered 1% formalin under the plantar surface of the right hind paw of mice after acute or intermittent fasting. Formalin-induced licking behaviors were measured for 40 min at 5-min intervals (Fig. 1a). Consistent with previous reports, 6 h acute fasting (AF 6 h) induced a significant analgesic effect on the second phase of nociceptive behaviors compared to the control group (Fig. 1b; *p < 0.05 vs. control group). Interestingly, more significant analgesic effects were showed in 12 h acute fasting (AF 12 h), 24 h acute fasting (AF 24 h), 12 h intermittent fasting (IF 12 h), and 24 h intermittent fasting (IF 24 h) groups (Fig. 1b; ***p < 0.0001 vs. control group). These results indicated that acute and intermittent fasting might have an analgesic effect on the 2nd phase of formalin-induced inflammatory pain.

### Acute or intermittent fasting has no effect on motor function

**Figure 2:**
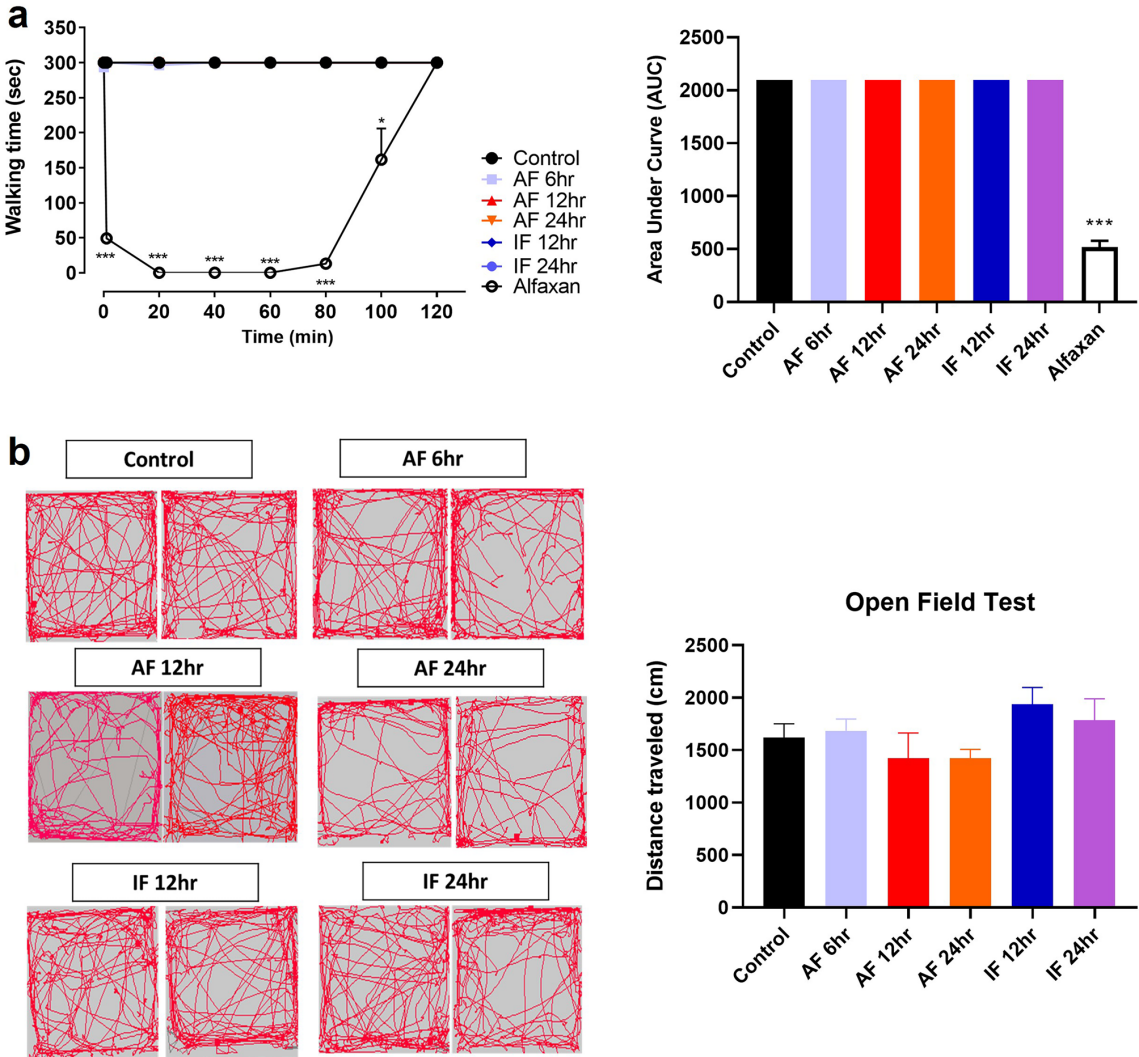
Effects of acute and intermittent fasting on motor function. (**a**) Rota-rod test was performed after 6 h acute fasting (AF 6 h), 12 h acute fasting (AF 12 h), 24 h acute fasting (AF 24 h), 12 h intermittent fasting (IF 12 h), 24 h intermittent fasting (IF 24 h), and alfaxan administration (positive control). Area under curve (AUC) was analyzed in control, fasting, and alfaxan-treated groups. (**b**) Open field test was performed in control and fasting groups and the distance traveled for 5 min was analyzed and plotted in a graph. Data were expressed as the mean ± SEM. *p < 0.05 and ***p < 0.0001 vs. Control. n = 5 mice/group.

The rota-rod and open field tests were performed to examine the changes in motor function after acute and intermittent fasting. Walking time on a rotating rod was measured and analyzed in control, acute fasting, intermittent fasting, and alfaxan-treated groups. There was no difference between control and acute (AF 6 h, AF 12 h, and AF 24 h) or intermittent (IF 12 h and IF 24 h) fasting groups, while alfaxan-treated group used as a positive control showed a significant reduction in performance time on a rotating rod (Fig. 2a; *p < 0.05, ***p < 0.0001 vs. control group). Next, mice were placed in the center of a square box and the moving distance was measured in the open field test. There was no difference in the distance traveled between control and acute (AF 6 h, AF 12 h, and AF 24 h) or intermittent (IF 12 h and IF 24 h) fasting groups (Fig. 2b). These results showed that the reduction of formalin-induced nociceptive responses in acute and intermittent fasting groups was not due to motor dysfunction.

### Fasting-induced changes in corticosterone levels and depression-like behavior

**Figure 3:**
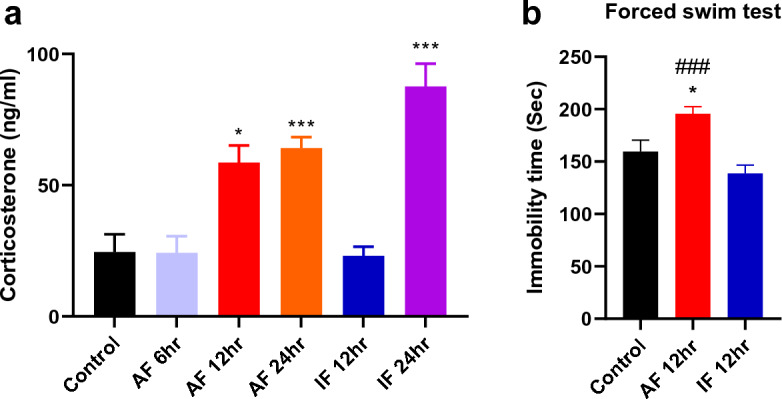
Effects of acute and intermittent fasting on the corticosterone levels in blood and the immobility time in forced swim test. (**a**) Corticosterone levels were measured after 6 h acute fasting (AF 6 h), 12 h acute fasting (AF 12 h), 24 h acute fasting (AF 24 h), 12 h intermittent fasting (IF 12 h), and 24 h intermittent fasting (IF 24 h) using ELISA. n = 4–7 mice/group. (**b**) Forced swim test was performed and immobility time (sec) was measured in control, 12 h acute fasting, and 12 h intermittent fasting groups. Data were expressed as the mean ± SEM. *p < 0.05 and ***p < 0.0001 vs. Control, ###*p* < 0.0001 vs. IF 12 h. n = 8–10 mice/group.

In order to identify the fasting duration that elicits analgesic effects without stress, we assessed corticosterone levels at different time intervals of fasting using ELISA. Interestingly, corticosterone levels in blood were significantly increased after 12 h acute fasting (AF 6 h), 24 h acute fasting (AF 24 h), and 24 h intermittent fasting (IF 24 h) compared with those of control group, whereas 12 h intermittent fasting (IF 12 h) had no effect on the levels of corticosterone (Fig. 3a; *p < 0.05, ***p < 0.0001 vs. control group). To determine whether the analgesic effects observed in the stress-programmed IF 12 h group and the stress-exposed AF 12 h group are associated with depression-like behavior, we conducted a forced swim test (FST). Immobility time in the forced swim test was significantly increased after 12 h acute fasting (AF 12 h) compared with that of control, while immobility time was not changed in the group with 12 h intermittent fasting (IF 12 h) (Fig. 3b; *p < 0.05 vs. control group, ###*p* < 0.0001 vs. IF 12 h). These results suggest that the analgesic effects observed in the AF 12 h group may be associated with elevated levels of corticosterone and depression-like behavior.

### Acute or intermittent fasting increases the activation of orexin neurons in the lateral hypothalamus

**Figure 4:**
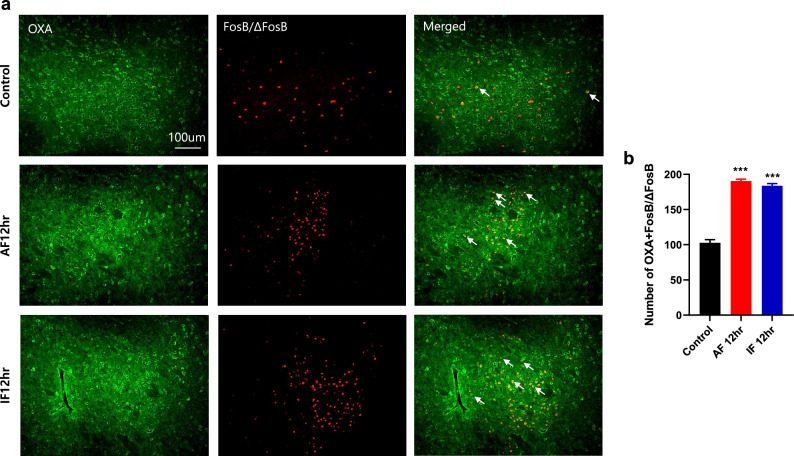
Effects of acute and intermittent fasting on the activation of orexin neurons in the lateral hypothalamus of mice. (**a**) Representative images of co-immunostaining with orexin A (OXA, green) and FosB/ΔFosB (red) in the lateral hypothalamus of control, 12 h acute fasting (AF 12 h), and 12 h intermittent fasting (IF 12 h) groups. White arrows indicate neurons that co-express orexin A and FosB/ΔFosB. (**b**) The number of cells co-expressing orexin A and FosB/ΔFosB was counted and shown as a graph. Data were expressed as the mean ± SEM. ***p < 0.0001 vs. Control. n = 5 mice/group.

To examine whether acute or intermittent fasting changes the activation of orexin neurons in the lateral hypothalamus (LH) after formalin administration, co-immunostaining was performed using specific antibodies for OXA and FosB/ΔFosB. Both 12 h acute fasting (AF 12 h) and 12 h intermittent fasting (IF 12 h) increased the number of cells co-expressing orexin A and FosB/ΔFosB in the lateral hypothalamus compared with that of control (Fig. 4a,b; ***p < 0.0001 vs. control group).

### Acute or intermittent fasting increases the expression of orexin A in the lateral hypothalamus and spinal cord, while the expression of orexin 1 receptor did not change in the spinal cord of mice

**Figure 5:**
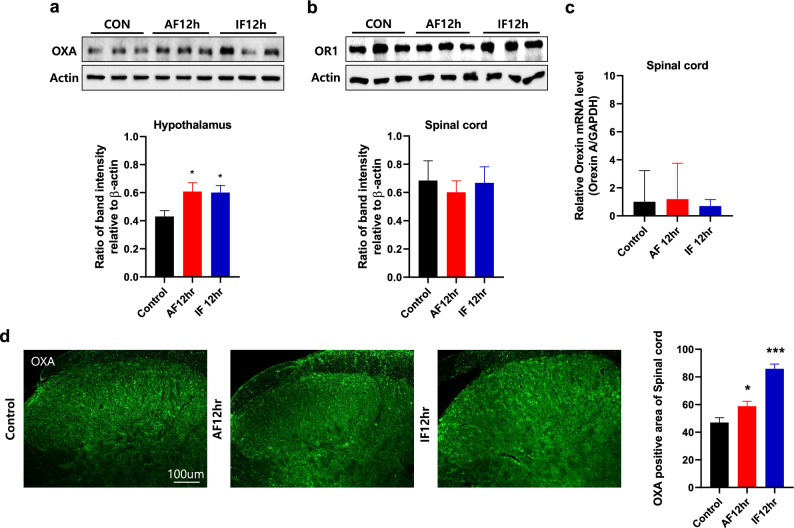
Effects of acute and intermittent fasting on the levels of protein and mRNA of orexin A or orexin 1 receptor in the hypothalamus and spinal cord of mice. (**a**) and (**b**) Western blot analysis was used to determine the expression levels of orexin A (OXA; **a**) and orexin 1 receptor (OR1; **b**) in the lateral hypothalamus of control, 12 h acute fasting (AF 12 h), and 12 h intermittent fasting (IF 12 h) groups. (**c**) OXA mRNA level was detected in the spinal cord of control, AF 12 h, and IF 12 h groups through qPCR. (d) Representative images showing OXA immunoreactivity in the spinal cord of control, AF 12 h, and IF 12 h groups. The changes in OXA immunoreactivity caused by fasting were shown as a graph. Data were expressed as mean ± SEM. WB was quantified as actin and qPCR as GAPDH. *p < 0.05 and ***p < 0.0001 vs. Control. n = 4–11 mice/group.

Next, we examined whether the expression of OXA or orexin 1 receptor (OR1) is changed in the lateral hypothalamus and spinal cord by acute or intermittent fasting. The expression of OXA was significantly increased in the lateral hypothalamus in the group with AF 12 h (*p < 0.05 vs. control group) or IF 12 h (*p < 0.05 vs. control group) compared with that of control group (Fig. 5a). Neither 12 h acute fasting nor 12 h intermittent fasting had an effect on the expression of OR1 in the spinal cord (Fig. 5b). The level of orexin A mRNA did not change in the spinal cord of mice after 12 h acute fasting or 12 h intermittent fasting (Fig. 5c). On the other hand, OXA immunoreactivity was increased in the spinal cord of AF 12 h (*p < 0.05 vs. control group) and IF 12 h (***p < 0.0001 vs. control group) groups compared with that of control group (Fig. 5d).

### Acute or intermittent fasting decreases the number of c-Fos-positive neurons in the spinal cord of mice after formalin administration

**Figure 6:**
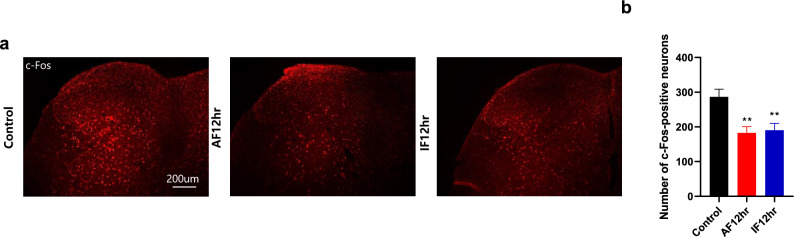
Effect of acute and intermittent fasting on the number of c-Fos-positive neurons after formalin administration. (**a**) Representative images showing c-Fos-positive neurons number in the dorsal horn lamina 1 and 2 of the L4-6 spinal cord in groups of control, 12 h acute fasting (AF 12 h), and 12 h intermittent fasting (IF 12 h) groups. (**b**) The c-Fos-positive neurons number in the dorsal horn lamina 1 and 2 shown in (a) was quantified and graphed. Data were expressed as mean ± SEM. **p < 0.01 vs. Control. n = 4–5 mice/group.

To investigate whether fasting modulates neuronal activation in the spinal cord after formalin administration, we examined the number of c-Fos-positive neurons in the spinal cord lamina 1 and 2 of acute or intermittent fasting group 3 h after formalin injection. Number of c-Fos-positive neurons was significantly decreased in the dorsal horn region lamina 1 and 2 of the L4-6 spinal cord in AF 12 h and IF 12 h (**p < 0.01 vs. control group) compared with that of control group (Fig. 6a,b).

### Blockade of spinal orexin 1 receptor with SB334867 reverses the inhibitory effect of intermittent fasting on formalin-induced nociceptive behaviors and c-Fos expression

**Figure 7:**
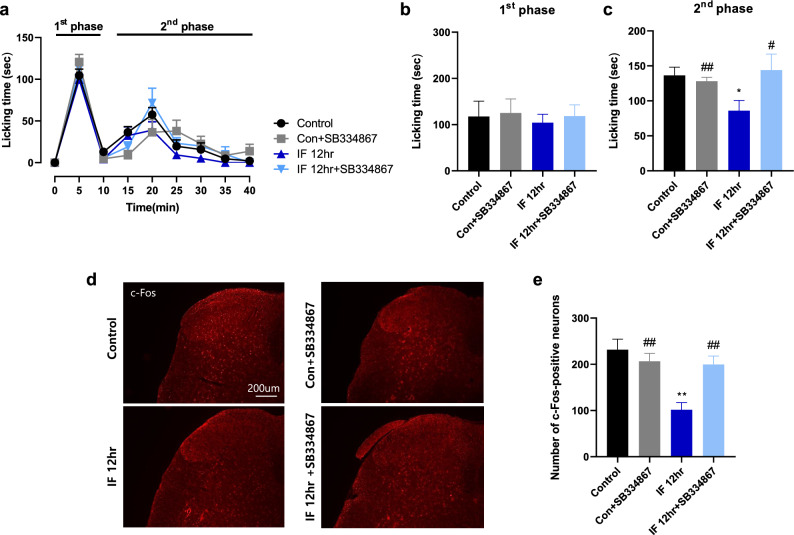
Effects of intrathecal SB334867 administration on formalin-induced nociceptive behaviors and spinal c-Fos expression after intermittent fasting of mice. (**a**) Formalin-induced nociceptive behaviors were measured in groups of vehicle-treated control, SB334867-treated control, vehicle-treated intermittent fasting (IF), and SB334867-treated IF groups. (**b**) and (**c**) The spontaneous nociceptive behaviors were divided into 1st phase (0–10 min) and 2nd phase (10–40 min). Intrathecal injection of SB334867 had no effect on licking behaviors during the 1st phase of pain (**b**), whereas formalin-induced licking behaviors during the 2nd phase of pain were reduced by SB334867 administration (**c**). (**d**) and (**e**) Representative images (**d**) and a graph (**e**) showing the effect of SB334867 on the number of c-Fos-positive neurons in the dorsal horn lamina 1 and 2 of the L4-6 spinal cord in control and IF groups. Data were expressed as mean ± SEM. *p < 0.05, **p < 0.01 vs. Control; #*p* < 0.05, ##*p* < 0.01 vs. IF 12 h. n = 5–8 mice/group.

To further investigate whether direct activation of orexin 1 receptor with OXA modulates nociceptive behaviors and spinal c-Fos expression after formalin administration, the specific OR1 antagonist, SB334867 was injected intrathecally 30 min prior to the administration of formalin in the paw. There were no significant changes in the time spent licking the formalin-injected paw during the first phase of pain (Fig. 7a,b). Intermittent fasting (IF) decreased the formalin-induced nociceptive behaviors during the second phase of pain (Fig. 7c; *p < 0.05 vs. control group), and this decrease was reversed by intrathecal administration of SB334867 in intermittent fasted mice (#*p* < 0.05 vs. IF 12 h group). Next, we examined the effect of OR1 activation on the number of c-Fos-positive neurons in the lumbar spinal cord dorsal horn of intermittent fasted mice. The number of c-Fos-positive neurons was decreased in the spinal cord lamina 1 and 2 of IF group (Fig. 7d,e; **p < 0.01 vs. control group), and this decrease was also restored by intrathecal SB334867 administration (##*p* < 0.01 vs. IF 12 h group). These results indicate that increased OXA expression in the spinal cord after intermittent fasting may play a role in antinociception against formalin-induced inflammatory pain via direct activation of OR1. The effects of SB334867 administration on formalin-induced nociceptive behaviors and spinal c-Fos immunoreactivity in acute fasted mice were shown in Supplementary Figure 1.

## Discussion

Up to now, our research has programmed animals to no longer feel stress by habituating them to fasting, commonly known as "stress fasting". To create groups that could be programmed, we first divided them into acute and intermittent fasting, with fasting periods of 12 and 24 h, respectively (with an additional 6 h for acute fasting). When subjected to the formalin test, a decrease in pain was observed during the 2nd phase of pain in all groups, similar to the previous research results that showed a reduction in pain behaviors in the group with 48 h fasting^18^. These results indicate that acute or intermittent fasting used in the present study is appropriate as an experimental model for stress fasting-induced analgesia.

When establishing our stress programming model, we measured plasma levels of corticosterone after acute or intermittent fasting. Although there are several hormones that can indicate stress, such as endorphins, adrenaline, and noradrenaline, we chose to focus on corticosterone, which is well-known and demonstrates analgesic effects through opioid neurons, similar to fasting. Fasting is perceived as a stressor in our body, leading to an increase in the levels of corticosterone, a biological marker of stress. However, when fasting is repeated at regular intervals, anticipated hunger (stress) responses are established, resulting in a stabilization of corticosterone levels. We confirmed the stress levels in each group using ELISA to establish stress-programmed groups based on corticosterone levels. Interestingly, acute or intermittent fasting revealed distinct temporal variations in the stress levels. Since the aim of the present study was to observe the analgesic effects of orexin A with reduced corticosterone levels, we selected intermittent fasting for 12 h, which induces hunger while maintaining low levels of corticosterone, as the representative group. In contrast, acute fasting for 12 h, which causes hunger and an increase in the levels of corticosterone, was chosen as the negative group. In the present study, 12 h intermittent fasting was not increased the levels of corticosterone, suggesting that this stress was programmed and habituated.

The induction of FosB/ΔFosB protein is longer lasting than c-Fos (approximately peaked at 3–6 h post stimulation)^19^ and some researchers have employed antibodies specific to a truncated isoform of accumulated ΔFosB protein in response to repetitive and chronic stimuli^20–24^. Consistent with previous research indicating that orexin neurons in the LH are activated following fasting, we observed an increase in FosB/ΔFosB immunoreactivity, merged with OXA, in animals subjected to fasting. This result suggests that OXA neurons are activated in the LH of fasted animals and that animals feel hungry when undergoing fasting. When orexin neurons are activated in the LH, OXA is widely projected throughout the brain via axons to post-synaptic target areas^25^. Therefore, our results, which showed the activation of OXA neurons in the LH, suggest the possibility that OXA has already been widely projected. In addition, our confirmation of increased OXA protein in the LH following fasting is consistent with other studies, which support the establishment of a stress programming model where corticosterone does not increase and OXA increases, as evidenced by molecular data.

The spinal cord is an important center for pain modulation, and the dorsal horn is a critical area for the transmission and integration of peripheral nociceptive signals^26^. The hypothalamic orexin neurons send their axons throughout laminae I-II of the dorsal horn, with OXA being most abundant in these two laminae^27^. Studies have found that intrathecal administration of OXA can significantly reduce behavioral responses to thermal and mechanical pain in rats, suggesting a potential role of orexin in sensation and pain modulation^28^. Therefore, we considered orexin that ascends from the spinal cord to be important and conducted staining to confirm its increase after fasting. Our results showed increased immunoreactivity for orexin in the fasting group, leading us to believe that the increased orexin may regulate pain. However, since protein and mRNA levels of OXA did not differ significantly from those of control group, we interpreted these results as indicating that the increased orexin was received via long axons from the LH, consistent with previous studies^29^. To assess the pain modulation capability of the increased orexin in the spinal cord, we injected formalin into the paw and confirmed c-Fos immunoreactivity three hours later. We interpreted the observed decrease in c-Fos immunoreactivity after fasting as indicating that the increased orexin expression reduced c-Fos activity and may regulate pain following formalin administration.

Neurons in the lateral hypothalamus and periventricular regions produce the neuropeptide OXA, which projects to various areas of the brain, including the spinal cord and olfactory bulb^30^. Orexin receptors are G protein-coupled receptors (GPCRs), and thus are expected to utilize heterotrimeric G proteins as major mediators of signal transduction^31, 32^. G proteins are known to have predominantly excitatory effects. However, affinity for OXA is expressed in the dorsal root ganglia (DRG) and spinal cord white matter^33^, and OXA modulates nociceptive reception by inhibiting Ca^2+^ influx via L-type Ca^2+^ channels^34^. Activation of the OXA-OR1 pathway may be more effective in inducing long-term potentiation in laminae I and II neurons by regulating synaptic transmission of TRPV1-positive C fibers^35^. However, it should be noted that OXA also has analgesic effects in other types of chronic pain. Specifically, intrathecal administration of OXA has been reported to have analgesic effects in the hot plate test and decrease the number of neurons in layers I and II with Fos-like immunoreactivity in the dorsal horn^36^. In addition to the potential analgesic mechanism of OXA mentioned above, one study demonstrated that chronic pain and specific stress conditions activate orexin neurons and suppress pain transmission^37^. Taken together with previous literature, it can be shown that although OR1 is a GPCR, it can suppress pain transmission, which is consistent with our formalin test results and c-Fos staining results.

In conclusion, this study revealed that increased levels of OXA in the spinal cord of chronically food-restricted mice programmed with stress reduce acute pain induced by formalin administration. Previous studies have shown that pain reduction in food-restriction models is mediated by stress-related hormone pathways. However, our study demonstrated the role of increased OXA exclusively, as we habituated the mice to stress without inducing the release of the stress hormone corticosterone. These findings show great potential for future studies on pain control mechanisms focusing on the OXA-centered pathway and suggest significant implications for developing innovative therapeutic strategies for pain management.

## Methods

### Animals

All experiments were approved by Chungnam University Medical College Research Ethics Committee, Korea. Adult male ICR mice (28–35 g) were purchased from DBL (Daehan Biolink, Korea) and Samtako Bio (Samtako Bio Inc, Korea). Animals were kept in temperature- and humidity-controlled room (22 ± 2 °C, 30–60%) with a 12 h light–dark cycle. All mice were acclimated at a local animal facility for a consistent duration of 1 week prior to the commencement of the experiments. The experimental protocols for animal usage were reviewed and approved by the CNU Animal Care and Use Committee (approve number: 202209A-CNU-157) and conform to NIH guidelines (NIH publication no.86–23, revised 1985). This study was carried out in accordance with the ethical guidelines for investigations of experimental pain in conscious animals^38^. This study is reported in accordance with ARRIVE guidelines.

### Food divestment

Acute fasting (AF) groups were withdrawn food 6, 12 and 24 h prior to performing the formalin test, and intermittent fasting (IF) groups were fasted (eating/fasting) for 12 or 24 h starting one week prior to the experiment. Water was freely available in fasting groups. Control group had free access to food and water.

### Formalin test

Mice were acclimatized for 30 min in an acrylic observation chamber (40 cm height and 20 cm diameter) before formalin injection. Formalin (1% in physiological saline, 20 μl) was injected subcutaneously into the plantar surface of the right hind paw using a Hamilton syringe connected to a 30-gauge needle. Then mice were returned to the observation chamber and formalin-induced nociceptive behaviors were recorded for 40 min. The amount of time the animal spent licking the injected paw was analyzed in two phases: 1st phase (0–10 min) and 2nd phase (10–40 min). Mice displaying atypical behavior following footpad injection of formalin were excluded from the analysis.

### Rota-rod test

Rota-rod test was performed to evaluate the effect of fasting on motor performance. All mice were trained to walk on a rota-rod apparatus (SciTech Korea Inc., Seoul, Korea) for 3 days before the initiation of experiments. Each mouse was placed on a rotating rod at a speed of 10 rpm for 5 min and the walking time (sec) was measured. After 15-min break, the test was performed again and repeated for a total of 120 min.

### Open field test

Open field test was conducted to confirm whether there was a behavioral disorder in mice under fasting conditions. Mice were placed in the center of a black square arena (40 × 40 cm), and their movement distance (cm) was monitored for 5 min following a 45-s adaptation period. The total distance traveled by the mice was analyzed using the EthoVision XT 11.5 program.

### Forced swim test

Forced swim test was carried out in accordance with Porsolt's instructions with minor modifications. Mice were placed in a plexiglass cylinder (10 × 25 cm) filled with water (24 ± 0.5 °C, 15 cm depth) and forced to swim for 15 min. After 24 h from this pre-test, the mice were put in the identical setup for 6 min and attempted to escape. After the first minute of intense activity, the mice displayed a period of immobility during which they stopped swimming and climbing and floated still in the water, only moving their forelimbs slightly to maintain their heads above water. Immobility time (sec) was measured as the amount of time the mice remained motionless for the final five minutes of the testing period.

### Immunohistochemistry

Mice were deeply anesthetized at 3 h after formalin injection, and perfused with saline containing fixative (4% paraformaldehyde in PBS). Brain was then isolated immediately and fixed in the same fixative for 12 h. Using a cryotome (Leica, Germany), spinal cords (L_4-6_) and lateral hypothalamus (LH; range: 3.10 mm, bregma:  − 0.94 mm) were cut into frozen sections of 20 μm and 10 μm thickness, respectively. After washing the frozen sections with PBS and PBS containing 0.1% Triton X-100 solution, tissue sections were incubated with rabbit anti-orexin A antibody (1:500, AB3098, Millipore Sigma, USA), anti-FosB antibody (1:1000, ab11959, Abcam, UK, this antibody stained for both FosB and ΔFosB)^22^, or anti-c-Fos(1:1000, ab7963, Abcam, UK) antibody overnight at 4 °C. Sections were then incubated with rabbit anti-fluorescein isothiocyanate (FITC) antibody (1:500, Jackson ImmunoResearch, USA) or mouse anti-cyanine 3 (Cy3) antibody (1:500, Jackson ImmunoResearch, USA) for 90 min at room temperature. Finally, we applied mounting media containing DAPI (VECTORLABORATORIES). Individual sections were examined in the captured images using a microscope (Axio Scope A1; Zeiss, Germany) and a digital camera (Axiocam MRm; Zeiss, Germany). Analysis was performed using ImageJ program.

### Western blot assay

Mice were anesthetized with avertin (25 mg/kg), and then a part was isolated over the spinal cord. Protein was extracted from the spinal cord slice using a mixture of a protease inhibitor and Radioimmunoprecipitation assay (RIPA) lysis buffer. Protein concentration was determined by the Bicinchoninic acid (BCA) assay. 30 µg of each lysate was separated on a 10% sodium dodecyl sulfate (SDS)-polyacrylamide gel and transferred to a nitrocellulose membrane. The membrane was blocked with 5% skim milk mixture in 1 × tris-buffered saline with 0.1% Tween 20 (TBST) solution for 1 h at room temperature. The blot was then probed with primary antibodies specific for orexin A (1:1000, AB3098, Millipore Sigma, USA), orexin 1 receptor (1:1000, PA5-77566, invitrogen, USA), and ß-Actin (1:2000, A-5441, Millipore Sigma, USA) overnight at 4 °C. After washing the membranes were exposed to secondary antibodies, anti-rabbit antibody and anti-mouse antibody, for 1 h at room temperature. Proteins were visualized using an enhanced chemiluminescence substrate kit (ECL kit, advansta) and normalized to ß-Actin. Analysis was performed using ImageJ program.

### Real-time PCR

Mice were anesthetized by injection of avertin, and the spinal cord was extracted by pressure expulsion with air into an ice-cooled phosphate-buffered saline-filled glass dish and snap-frozen in liquid nitrogen. To verify the location of the L4-L6 spinal cord segments, the attachment site of each spinal nerve was identified in anesthetized mice. In addition, spinal segments were separated into left and right halves under a neurosurgical microscope. The spinal cord was subsequently further subdivided into dorsal and ventral halves by cutting straight across from the central canal laterally to a midpoint in the white matter. The right and left spinal dorsal horns were used for qPCR. Collected segments of the spinal cord were homogenized with TRIzol reagent (Thermo Fisher Scientific Inc., Waltham, MA, USA) for RNA extraction. The relative RNA expression was determined by a real-time polymerase chain reaction. Reaction conditions consisted of 10 min at 95 ℃, followed by 40 cycles of 15 s at 95 ℃, 20 s at 60 ℃, and 30 s at 72 ℃. Primers for mouse glyceraldehyde-3-phosphate dehydrogenase (GAPDH) were as follows: forward, 5’- AGGTCGGTGTGAACGGATTTG-3’; reverse, 5’- TGTAGACCATGTAGTTGAGGTCA-3’. Primers for mouse orexin A were as follows: forward, 5’- GTCGCCAGAAGACGTGTTC-3’; reverse, 5’- GGTGGTAGTTACGGTCGGAC-3’. Relative mRNA levels were calculated according to a comparative CT method using a reference gene.

### Blood sampling and corticosterone measurement

Blood was drawn from the heart of anesthetized mice and allowed to clot in serum tubes for 2 h at room temperature. Samples were centrifuged at 2000 g for 20 min. Corticosterone levels were investigated in serum samples using an ELISA kit (KGE009, R&D, USA). Absorbance was measured repeatedly at a wavelength of 450 nm using a plate reader (Sunrise microplate reader, TECAM, Spain).

### Intrathecal injection

Intrathecal administration was performed using a 20 μl Hamilton syringe connected to a 30-gauge needle, which was inserted at a 45° angle to the vertebral column into the intervertebral space between the spinous process of L5-6. SB334867, a selective orexin 1 receptor antagonist (3 nmol in 5% DMSO), or vehicle (5% DMSO) was progressively each 10 μl injected, and the syringe was kept for a further 10 s before being removed to stop the medication from dispensing. Intrathecal injection was performed 30 min prior to the administration of formalin in the paw.

### Statistical analysis

GraphPad Prism (GraphPad Software, USA) was used for statistical analysis. The data were presented as the mean ± SEM. The degree of statistical significance was established using an unpaired Student’s t-test for comparisons between two means and an analysis of variance (ANOVA) followed by a Dunnett’s test for multiple comparisons.

## Supplementary Information

Supplementary Information.

## Data Availability

Data are available on request from the corresponding author.
