# Author Correction: Antinociceptive effect of intermittent fasting via the orexin pathway on formalin-induced acute pain in mice

**DOI:** 10.1038/s41598-025-07149-5

**Published:** 2025-06-20

**Authors:** Hyunjin Shin, Jaehyuk Kim, Sheu-Ran Choi, Dong-Wook Kang, Ji-Young Moon, Dae-Hyun Roh, Miok Bae, Jungmo Hwang, Hyun-Woo Kim

**Affiliations:** 1 Department of Physiology and Medical Science, College of Medicine and Brain Research Institute, Chungnam National University, 266 Munhwa-ro, Jung-gu, Daejeon, 35015 Korea; 2 CNS Team, N-DIC, Hwaseong, 18469 Korea; 3 Department of Pharmacology, Catholic Kwandong University College of Medicine, Gangneung, 25601 Korea; 4 Animal Protection and Welfare Division, Animal and Plant Quarantine Agency, Gimcheon, 39660 Korea; 5 Department of Oral Physiology, School of Dentistry, Kyung Hee University, Seoul, 02447 Korea; 6 Preclinical Research Center, Chungnam National University Hospital, Daejeon, 35015 Korea; 7 Department of Orthopaedic Surgery, College of Medicine, Chungnam National University, 266 Munhwa-ro, Jung-gu, Daejeon, 35015 Korea

Correction to: *Scientific reports* 10.1038/s41598-023-47278-3, published online 20 November 2023

The original version of this Article contained an error in Figure 2, panel b, where two identical images were used in the open field trace data of “AF 24hr”. The original Figure 2 and accompanying legend appear below.

**Fig. 2:**
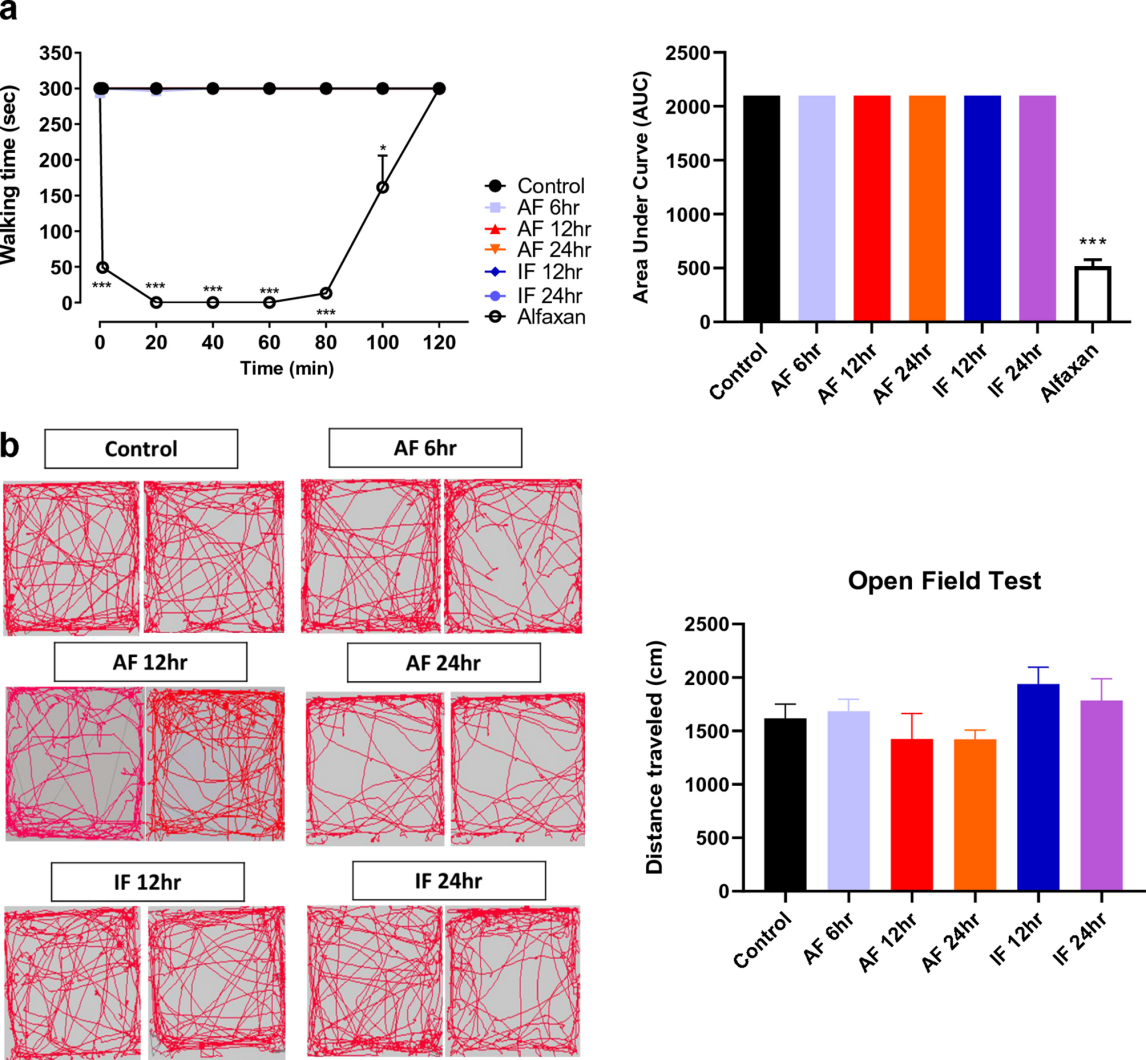
Effects of acute and intermittent fasting on motor function. (**a**) Rota-rod test was performed after 6 h acute fasting (AF 6 h), 12 h acute fasting (AF 12 h), 24 h acute fasting (AF 24 h), 12 h intermittent fasting (IF 12 h), 24 h intermittent fasting (IF 24 h), and alfaxan administration (positive control). Area under curve (AUC) was analyzed in control, fasting, and alfaxan-treated groups. (**b**) Open field test was performed in control and fasting groups and the distance traveled for 5 min was analyzed and plotted in a graph. Data were expressed as the mean ± SEM. *p < 0.05 and ***p < 0.0001 vs. Control. n = 5 mice/group.

The original Article has been corrected.

