# Supplementary material for: Antinociceptive effect of intermittent fasting via the orexin pathway on formalin-induced acute pain in mice
Source: Sci Rep. 2023 Nov 20;13:20245. doi: 10.1038/s41598-023-47278-3 (PMC10661460; doi:10.1038/s41598-023-47278-3)
Supplement: Supplementary file 1 — Supplementary Information. [file 41598_2023_47278_MOESM1_ESM.pdf]

Supplementary data

Figure5. Western blot

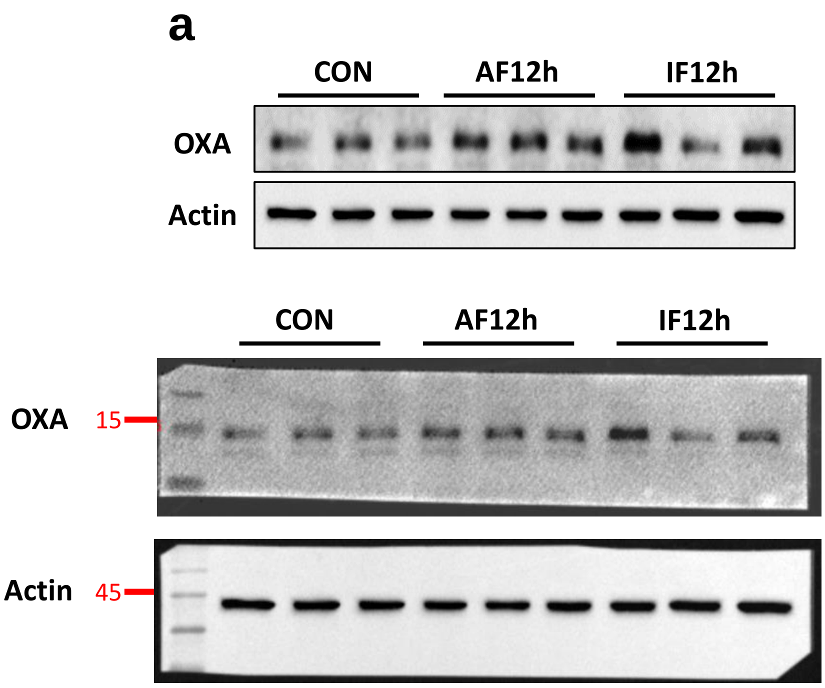

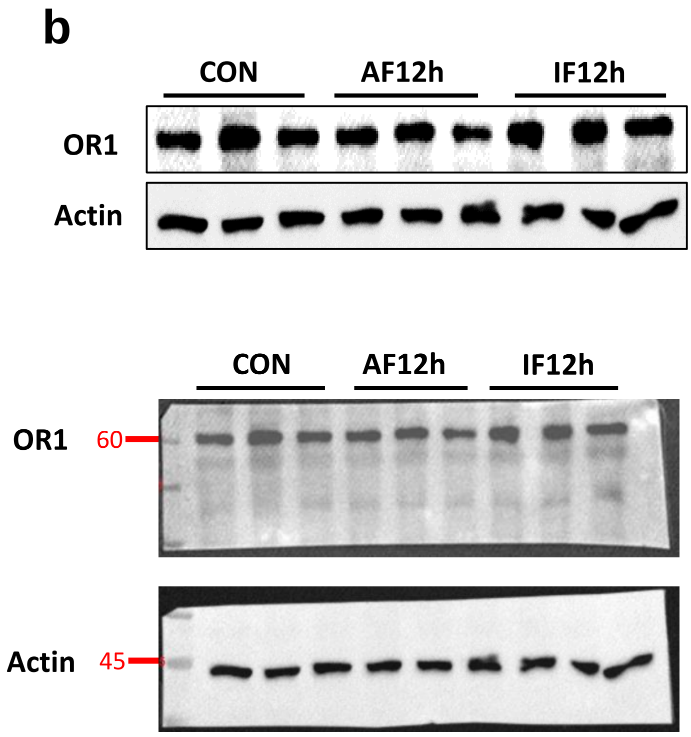

Supplementary Figure1.

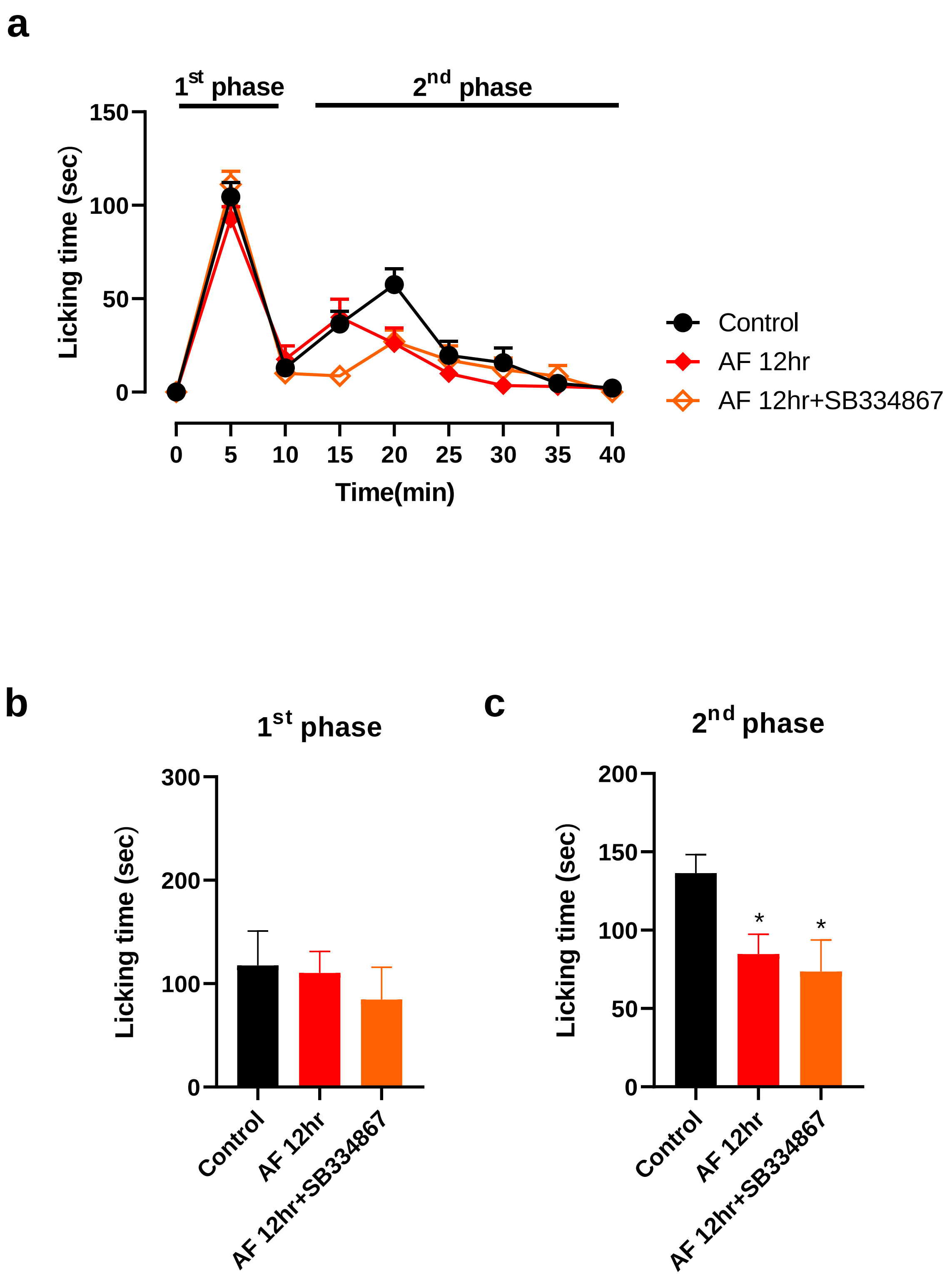

d

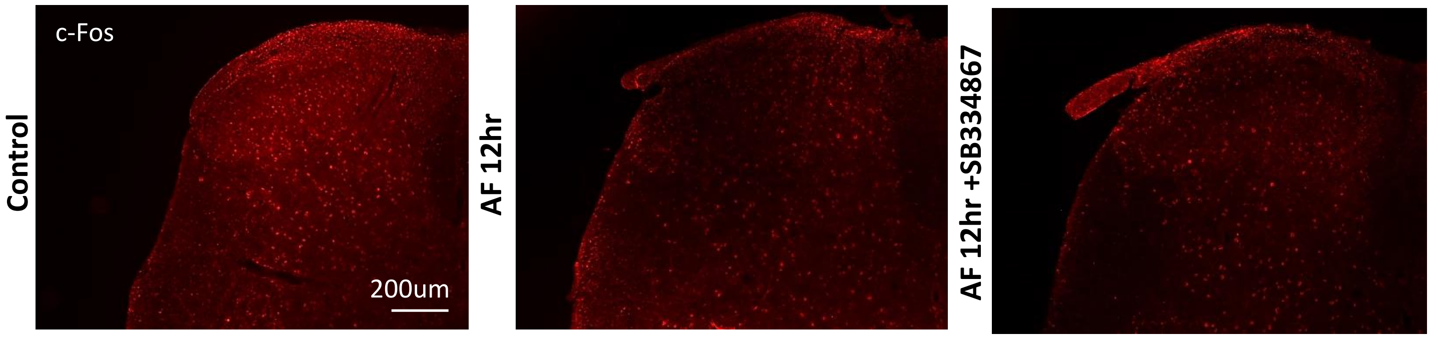

Control
c-Fos
200um
AF 12hr
AF 12hr +SB334867

e

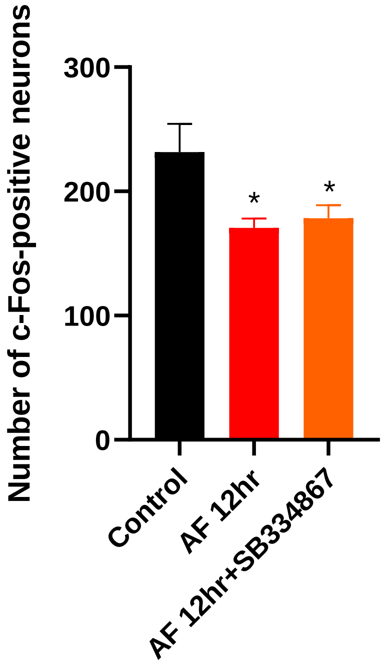

Number of c-Fos-positive neurons
300
200
100
0
*
*
Control
AF 12hr
AF 12hr+SB334867

Supplementary data 1 legend

Effects of intrathecal SB334867 administration on formalin-induced nociceptive behaviors and spinal c-Fos expression after acute fasting of mice.

(**a**) Formalin-induced nociceptive behaviors were measured in groups of control, vehicle-treated acute fasting (AF) 12 hr, and SB334867-treated AF 12 hr groups. (**b** and **c**) The spontaneous nociceptive behaviors were divided into 1st phase (0–10 min) and 2nd phase (10–40 min). Intrathecal injection of SB334867 had no effect on licking behaviors during the 1st phase of pain (**b**). Formalin-induced licking behaviors during the 2nd phase of pain were also unaffected by SB334867 administration (**c**). (**d** and **e**) Representative images (**d**) and a graph (**e**) showing the effect of SB334867 on the number of c-Fos-positive neurons in the dorsal horn lamina 1 and 2 of the L4-6 spinal cord in control and AF groups. Data were expressed as mean ± SEM. * $p<0.05$ vs. Control. n = 5-8 mice/group.

Supplementary data 1.

To investigate whether fasting modulates neuronal activation in the spinal cord after formalin administration, we examined the number of c-Fos-positive neurons in the spinal cord lamina 1 and 2 of acute or intermittent fasting group 3 h after formalin injection. Consistent with Orexin A immunoreactivity in the spinal cord of the AF12hr group, there was an increase compared to the control group (Fig. 5). In the AF12hr + SB334867 group, the OR1 antagonist effectively inhibited the elevated OXA levels observed in the spinal cord of the AF12hr group, while there were no changes in formalin-induced pain. Similarly, no alterations were observed in the number of c-Fos-positive neurons in both the AF12hr group and the AF12hr + SB334867 group. These results collectively suggest that the analgesic effect observed in the AF12hr group is influenced more by stress-induced analgesia than by the orexin A pathway.
